# Environmental contamination in a coronavirus disease 2019 (COVID-19) intensive care unit—What is the risk?

**DOI:** 10.1017/ice.2020.1278

**Published:** 2020-10-21

**Authors:** Sean Wei Xiang Ong, Pei Hua Lee, Yian Kim Tan, Li Min Ling, Benjamin Choon Heng Ho, Ching Ging Ng, Dong Ling Wang, Boon Huan Tan, Yee-Sin Leo, Oon-Tek Ng, Michelle Su Yen Wong, Kalisvar Marimuthu

**Affiliations:** 1 National Center for Infectious Diseases, Singapore; 2 Department of Infectious Diseases, Tan Tock Seng Hospital, Singapore; 3 DSO National Laboratories, Singapore; 4 Department of Respiratory & Critical Care Medicine, Tan Tock Seng Hospital, Singapore; 5 Lee Kong Chian School of Medicine, Nanyang Technological University, Singapore; 6 Yong Loo Lin School of Medicine, National University of Singapore, Singapore

## Abstract

**Background:**

The risk of environmental contamination by severe acute respiratory coronavirus virus 2 (SARS-CoV-2) in the intensive care unit (ICU) is unclear. We evaluated the extent of environmental contamination in the ICU and correlated this with patient and disease factors, including the impact of different ventilatory modalities.

**Methods:**

In this observational study, surface environmental samples collected from ICU patient rooms and common areas were tested for SARS-CoV-2 by polymerase chain reaction (PCR). Select samples from the common area were tested by cell culture. Clinical data were collected and correlated to the presence of environmental contamination. Results were compared to historical data from a previous study in general wards.

**Results:**

In total, 200 samples from 20 patient rooms and 75 samples from common areas and the staff pantry were tested. The results showed that 14 rooms had at least 1 site contaminated, with an overall contamination rate of 14% (28 of 200 samples). Environmental contamination was not associated with day of illness, ventilatory mode, aerosol-generating procedures, or viral load. The frequency of environmental contamination was lower in the ICU than in general ward rooms. Eight samples from the common area were positive, though all were negative on cell culture.

**Conclusion:**

Environmental contamination in the ICU was lower than in the general wards. The use of mechanical ventilation or high-flow nasal oxygen was not associated with greater surface contamination, supporting their use and safety from an infection control perspective. Transmission risk via environmental surfaces in the ICUs is likely to be low. Nonetheless, infection control practices should be strictly reinforced, and transmission risk via droplet or airborne spread remains.

The coronavirus disease 2019 (COVID-19) pandemic has spread at an exponential rate since the first recognition of the novel virus, severe acute respiratory syndrome coronavirus 2 (SARS-CoV-2), and has placed a disproportionate strain on intensive care resources worldwide.^1,2^

Healthcare facilities have been implicated as centers of transmission, in both tertiary-care hospitals and long-term care facilities.^3-7^ However, the frequency of nosocomial transmission in ICUs is less clear, and no large ICU outbreaks have been reported to date. ICUs have been important sites of nosocomial transmission and super-spreading events in previous coronavirus outbreaks, in part due to the increased frequency of use of aerosol-generating procedures (AGPs), particularly endotracheal intubation.^8,9^

Other noninvasive oxygenation strategies, such as positive pressure noninvasive ventilation (NIV) or high-flow nasal oxygen (HFNO), have been shown to be beneficial in reducing mortality and progression to intubation in hypoxemic respiratory failure.^10^ However, from an infection control perspective, these strategies are AGPs with an increased risk of aerosol transmission and environmental contamination via droplet dispersion. The extent of transmission risk through environmental contamination from these procedures remains unclear, and recommendations from different regulatory authorities have varied in their definition of AGP and their relative risk.^11^

Extensive environmental contamination by SARS-CoV-2 in the environments of infected patients has been demonstrated in multiple studies in both healthcare and community settings,^12-20^ but no study has focused specifically on the extent of such contamination in an ICU setting nor correlated patient and disease factors with the extent of environmental contamination, including the impact of ventilation modalities. A study found that environmental contamination decreased sharply after day 7 of illness,^13^ which was hypothesized to be related to the similar decrease in viral load from the upper respiratory tract in the same time frame.^21-23^

In this study, we evaluated the extent of environmental contamination by SARS-CoV-2 in an ICU setting and correlated these findings with patient and disease factors to assess the relative safety of different oxygenation methods with regard to environmental contamination. We hypothesized that despite the increased use of noninvasive ventilatory methods, environmental contamination in the ICU would be lower (1) because most COVID-19 patients typically deteriorate in the second week of illness, during which period viral shedding decreases^24,25^ and (2) because closed-loop ventilatory circuits contain and limit the spread of contaminating droplets or aerosols. Additionally, we conducted a literature review to assess the extent and frequency of ICU environmental contamination across different healthcare systems worldwide.

## Methods

### Collection of environmental samples

This study was conducted in 2 dedicated COVID-19 ICUs in the National Centre for Infectious Diseases, the largest outbreak center for COVID-19 in Singapore. These ICUs admitted both patients with confirmed COVID-19 requiring intensive care as well as suspected patients with respiratory symptoms undergoing evaluation to rule out COVID-19. Environmental sampling was carried out at 5 separate time points in the rooms of all patients with active COVID-19 infection, defined by a positive SARS-CoV-2 polymerase chain reaction (PCR) test from any respiratory sample. Patients were housed in single airborne infection isolation rooms (AIIRs) with attached anterooms. Patients who had ceased viral shedding (ie, latest respiratory sample was negative for SARS-CoV-2 PCR) were excluded. In total, 10 sites were sampled from each room (Supplementary Fig. 1 online). In addition, 5 points in the common areas in the ICU were sampled, as well as 5 points in the staff pantry shared between both ICUs (Supplementary Fig. 2 online).

Environmental samples were collected by the same study team member throughout all sampling cycles using EnviroMax Plus premoistened macrofoam sterile swabs (Puritan Medical Products, Guilford, ME). The same surface area was swabbed for each sampling site using a standardized technique. This same environmental sampling protocol has been used in other studies at our center and has achieved consistent detection results.^12,13^ All samples were kept at 4°C and were transported to a biosafety level 3 (BSL-3) laboratory for storage and testing within 3 days of sampling.

### Clinical data collection

Clinical data including day of illness, type of oxygenation or ventilatory support, use of AGPs (intubation, extubation, open suctioning, nebulization, or bronchoscopy), and clinical cycle threshold (Ct) value (if available) were collected from the electronic medical record using a standardized case report form. No patient identifiers were recorded, and data were stored on a secured server. Informed consent was waived as clinical data were collected as part of an outbreak investigation under the Infectious Diseases Act, authorized by the Ministry of Health, Singapore.

### Cleaning regimen of rooms

Routine twice-daily environmental cleaning in the ICU rooms was performed by housekeeping staff, using 5,000 parts per million (ppm) sodium dichloroisocyanurate (NaDCC) for environmental surfaces and 1,000 ppm NaDCC for the floor. Cleaning of common areas was also performed twice daily with 1,000 ppm NaDCC for the floor and high-touch surfaces. All environmental sampling was conducted in the morning before the scheduled environmental cleaning (ie, the last cleaning time was the afternoon prior to environmental sampling).

### Polymerase chain reaction methods

Sample RNA extraction was performed using the QIAamp viral RNA mini kit (Qiagen, Hilden, Germany) according to the manufacturer’s instructions. Real-time PCR assays targeting the envelope (E) gene^26^ and orf1ab assay adapted from Drosten et al^27^ were used for the detection of SARS-CoV-2 RNA. For the E gene assay, a 20 µL reaction mix was prepared with 12.5 µL of SuperScript III Platinum One-Step qRT-PCR Kit (Thermofisher Scientific, Waltham, MA) buffer, 0.75 mM Mg_2_SO_4_, 5 µL of RNA, 400 nM each of the forward primer (E_Sarbeco_F1-ACAGGTACGTTAATAGTTAATAGCGT) and reverse primer (E_Sarbeco_R2-ATATTGCAGCAGTACGCACACA) with 200 nM of probe (E_Sarbeco_P1-(FAM) ACACTAGCCATCCTTACTGCGCTTCG (BHQ1)). Thermal cycling conditions included reverse transcription at 55°C for 10 minutes, an initial denaturation at 95°C for 5 minutes, followed by 45 cycles of 95°C for 15 seconds, 58°C for 1 minute. For the orf1ab assay, a 20 µL reaction mix was prepared with 12.5 µL SuperScript III Platinum One-Step qRT-PCR Kit buffer, 0.5 mM Mg_2_SO_4_, 5 µL RNA, 800 nM each of the forward primer (Wu-BNI-F-CTAACATGTTTATCACCCGCG) and reverse primer (Wu-BNI-R-CTCTAGTAGCATGACACCCCTC) with 400 nM of probe (WU-BNI-P-(FAM) TAAGACATGTACGTGCATGGATTGGCTT (BHQ1)). Thermal cycling conditions included reverse transcription at 55°C for 10 minutes, an initial denaturation was conducted at 95°C for 5 minutes, followed by 45 cycles of 95°C for 15 seconds and 60°C for 1 minute. All samples were run in duplicate and with both assays with positive and negative controls with each sample run. Positive detection was recorded as long as amplification was observed in at least 1 assay.

### Virus culture methods

Positive swabs by PCR from the common area and staff pantry were further evaluated for virus viability via cell culture. Monolayers of Vero C1008 cells (ATCC-1586) in T25 flasks were inoculated with 1 mL inoculum (500 µL of the swab sample and 500 µL of Eagle’s MEM) and cultured at 37°C, 5% CO_2_ with blind passage every 7 days. Also, 140 µL cell culture was used for RNA extraction and real-time PCR twice per week to monitor changes in target SARS-CoV-2 genes as an indication of successful viral replication. In the absence of cytopathic effects and real-time PCR indication of viral replication, blind passages continued for a total of 4 passages before any sample was determined to be negative of viable SARS-CoV-2 virus particles.

### Statistical analysis

Extent of ICU contamination was compared with previously published historical data from 30 rooms (27 general ward and 3 ICU) from our center.^13^ Categorical variables were compared using the Fisher exact test, and continuous variables were compared using the Mann-Whitney U test. Binary logistic regression analysis was used to determine odds ratios (ORs) and 95% confidence intervals (CIs) for variables associated with presence of environmental contamination. *P* < .05 was considered significant, and all tests were 2-tailed. Analyses were performed using Stata version 13 software (StataCorp, College Station, TX).

### Literature review

Other studies evaluating environmental contamination of hospital environments by SARS-CoV-2 were analyzed and compared in relation to our study results. We searched PubMed for manuscripts in English published before July 19, 2020, using varying combinations of the search terms “environmental,” “contamination,” “SARS-CoV-2,” “COVID-19,” and “hospital.” All manuscripts that reported results of environmental sampling in hospital environments were included and results were extracted and compared.

## Results

### Sample collection and clinical data

In total, 200 samples from 20 patient rooms were collected across 5 sampling time points; 60 samples from the ICU common areas were collected across 3 sampling time points; and 15 samples from the staff pantry were collected across 3 sampling time points. Of the 20 patients whose rooms were sampled, the median age was 51.5 years old (interquartile range [IQR] 39–67.75), and 15 (75%) were men. The median day of illness was day 14 (IQR, 9.25–18.75), and the median clinical Ct value was 31.22 (IQR, 27.31–34.56; n = 18 because 2 patients had only qualitative PCR reported).

Moreover, 7 patients (35%) were intubated and mechanically ventilated, 9 (45%) were receiving HFNO, and 4 (20%) did not receive any supplementary oxygen or ventilatory support. These last 4 patients were in the ICU for closer monitoring for other nonrespiratory complications (eg, myocardial infarction or arrhythmias requiring cardiac monitoring). Also, 3 patients (15%) had AGPs performed within the 24 hours prior to sampling.

### Contamination of patient rooms

**Fig. 1.**
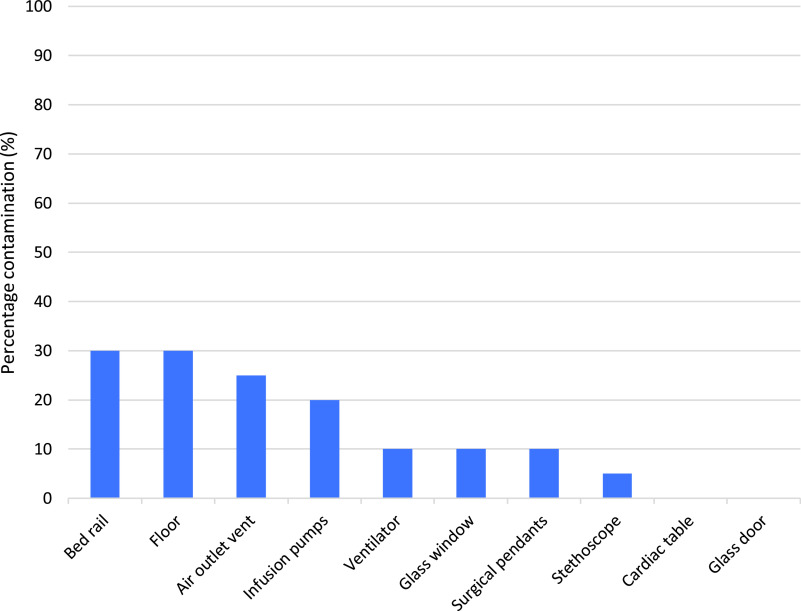
Percentage contamination by sites sampled in patient rooms.

Overall, 14 rooms had at least 1 site that was contaminated (median number of sites, 2; IQR, 1–2; range 1–5) (Table 1). The most frequently contaminated sites were the bed rail and floor (30%), followed by the air outlet vent (25%) and infusion pumps (20%) (Fig. 1). Presence of environmental contamination was not significantly associated with age, sex, day of illness, ventilatory mode, AGP, or clinical Ct value (Table 2). Contamination was identified in rooms with patients on mechanical ventilation, HFNO, as well as those not requiring any ventilatory support. Viral cell culture was not attempted on patient room samples due to resource limitations.

**Table 1.** Clinical Data of Patients in Rooms Sampled and Sites of Environmental Contamination

No.	Age	Sex	Day of Illness	Ventilatory Support	AGP in the Past 24 h	Clinical Ct Value	Environmental Contamination	Percentage Contamination	Location									
									CT	BR	VT	IP	ST	FL	GW	GD	AV	SP
1	50	F	19	HFNO	Nil	34.38	Yes	10	…	X	…	…	…	…	…	…	…	…
2	64	M	16	HFNO	Extubation	34.40	Yes	20	…	X	…	X	…	…	…	…	…	…
3	61	M	20	HFNO	Extubation	33.50	Yes	10	…	X	…	…	…	…	…	…	…	…
4	36	F	18	HFNO	Nil	35.48	No	0	…	…	…	…	…	…	…	…	…	…
5	86	F	32	Intubated	Nil	35.04	Yes	10	…	…	…	…	…	…	X	…	…	…
6	82	M	14	Intubated	Nil	27.77	Yes	10	…	…	…	…	…	…	…	…	X	…
7	69	F	10	Intubated	Nil	23.31	Yes	30	…	…	…	…	X	X	…	…	…	X
8	60	M	23	Intubated	Nil	23.13	No	0	…	…	…	…	…	…	…	…	…	…
9	37	M	21	Nil	Nil	NA	Yes	10	…	…	…	…	…	X	…	…	…	…
10	73	M	12	Intubated	Intubation	29.55	No	0	…	…	…	…	…	…	…	…	…	…
11	52	M	9	HFNO	Nil	25.94	Yes	20	…	…	…	…	…	X	…	…	X	…
12	45	M	6	Nil	Nil	28.62	Yes	20	…	…	…	X	…	…	…	…	X	…
13	69	F	17	Intubated	Nil	32.50	No	0	…	…	…	…	…	…	…	…	…	…
14	51	M	11	HFNO	Nil	32.25	No	0	…	…	…	…	…	…	…	…	…	…
15	35	M	4	Nil	Nil	30.18	No	0	…	…	…	…	…	…	…	…	…	…
16	45	M	13	HFNO	Nil	38.99	Yes	20	…	…	X	…	…	X	…	…	…	…
17	52	M	16	Intubated	Nil	36.56	Yes	10	…	X	…	…	…	…	…	…	…	…
18	30	M	6	HFNO	Nil	24.34	Yes	20	…	X	…	…	…	X	…	…	…	…
19	35	M	14	Nil	Nil	NA	Yes	50	…	X	X	X	…	…	X	…	X	…
20	45	M	7	HFNO	Nil	29.93	Yes	40	…	…	…	X	…	X	…	…	X	X

Note. AGP, aerosol-generating procedure; Ct, cycle threshold; CT, cardiac table; BR, bed rail; VT, ventilator; IP, infusion pumps; ST, stethoscope; FL, floor; GW, glass window; GD, glass door; AV, air outlet vent; SP, surgical pendant; F, female; M, male; HFNO, high-flow nasal oxygen; NA, not available; -, no contamination; X, contamination present

**Table 2.** Univariate Logistic Regression Analysis of Factors Associated With Presence of Environmental Contamination

Variable	Environmental Contamination (n=14)	No Environmental Contamination (n=6)	Odds Ratio (95% CI)	*P* Value
Age, median (IQR)	51 (45–64)	55.5 (36–69)	0.999 (0.94–1.06)	.98
Sex, male, no. (%)	11 (78.6)	4 (66.7)		.61
Day of illness, median (IQR)	14 (9–19)	14.5 (11–18)	1.01 (0.87–1.17)	.92
**Ventilatory method, no. (%)**				
Nil	3 (21.4)	1 (16.7)	Ref	Ref
Mechanical ventilation	4 (28.6)	3 (50)	0.44 (0.03–6.70)	.56
High-flow nasal oxygen	7 (50)	2 (33.3)	1.17 (0.07–18.35)	.91
AGP	2 (14.3)	1 (16.7)	0.83 (0.06–11.42)	.89
Clinical Ct value, median (IQR)	31.72 (26.86–34.72)	31.22 (29.55–32.50)	1.03 (0.83–1.27)	.81

Note. CI, confidence interval; IQR, interquartile range; ref, reference; Ct, cycle threshold; AGP, aerosol-generating procedure.

### Comparison of ICU and general ward contamination

Results from this study were compared to historical data from 27 general ward rooms and an additional 3 ICU rooms to assess the differences in environmental contamination between both settings. Both studies were conducted done at the same center and by the same study team; thus, the environmental sampling protocol and hospital environmental decontamination protocols were unchanged. Comparing all ICU rooms with all general ward rooms, although the proportion of rooms with any environmental contamination were similar, there appeared to be less contamination in the ICU, with a lower number of sites and percentages of sites contaminated (Table 3). However, due to possible confounding factors, tests were not performed to determine the statistical significance of this difference.

**Table 3.** Extent of Contamination in ICU Rooms Compared to General Ward Rooms^a,b^

Variable	All ICU Rooms (n=23), No. (%)	All General Ward Rooms (n=27), No. (%)
Day of illness, median (IQR)	14 (9–19)	7 (4–17)
Clinical Ct value, median (IQR)	30.18 (28.45–34.40)	30.40 (22.04–35.24)
Any environmental contamination (at least 1 site)	14 (60.9)	17 (63.0)
No. of sites contaminated, median (IQR)	1 (0–2)	7 (4–17)
% of sites contaminated, median (IQR)	10 (0–20)	14.3 (0–42.9)
	ICU Rooms With Contamination (n=14), No. (%)	General Ward Rooms With Contamination (n=17), No. (%)
No. of sites contaminated, median (IQR)	2 (1–2)	2 (1–5)
% of sites contaminated, median (IQR)	20 (10–20)	28.6 (14.3–62.5)

Note. ICU, intensive care unit; IQR, interquartile range; Ct, cycle threshold.

a 30 rooms (3 from ICU, 27 from general ward) were included from historical data in a previously published study for analysis to compare environmental contamination between ICU and general ward rooms.

b Categorical variables are expressed as number (percentage), continuous variables are expressed as median (IQR).

### Contamination of common areas and staff pantry

Of the 60 samples collected from the ICU ward common areas, 6 (10%) were positive for SARS-CoV-2: 5 samples from the floor and 1 sample from a desktop computer outside the patient room (Table 4). Of the 15 samples collected from the staff pantry, 2 (13.3%) were positive: 1 sample from the floor and 1 sample from a refrigerator door handle (Table 4). All samples were negative on viral cell culture.

**Table 4.** Results of Surface Sampling of Intensive Care Unit Common Areas and Staff Pantry

Surface Sampled	No. of Samples Collected	No. of Positive Samples	Cycle Threshold Value(s)	Viral Culture
**ICU ward common area**				
Floor	12	5	36.20–37.76	Negative
Nursing counter	12	0	…	…
Desktop computer	12	1	37.00	Negative
Mobile computer on wheels	12	0	…	…
PPE storage area	12	0	…	…
**Shared staff pantry**				
Floor	3	1	38.13	Negative
Sofa	3	0	…	…
Dining table	3	0	…	…
Water dispenser handle	3	0	…	…
Fridge door handle	3	1	38.14	Negative

### Literature review of environmental sampling studies

In total, 22 studies were identified that conducted environmental sampling of SARS-CoV-2. However, 2 studies were excluded because they were conducted outside of acute healthcare settings, 1 in a hotel quarantine facility and 1 in a community long-term care facility.^18,28^ Of the 20 remaining studies, 9 did not conduct any sampling in the ICU (Table 5). No study specifically focused on environmental contamination in the ICU, and the number of ICU samples ranged from 24 to 218 (median, 35; with 2 studies not stating the precise number of ICU samples). Percentage contamination of all environmental samples from ICU patient rooms ranged from 0 to 44%. Only 2 studies performed viral cultures, and these results were negative for all samples. Because sampling protocol, patient profile, and environmental set-ups differed greatly between studies, further statistical analyses were not performed to assess statistical differences between studies.

**Table 5.** Comparison of the Extent of Environmental Contamination in Hospital Environmental Sampling Studies

Study Author, Journal, Year	Total No. of Samples	Overall % Contaminated	ICU Sampling Done	No. of ICU Samples (Total)	No. of ICU Rooms Sampled	No. of ICU Room Samples	% Contaminated (ICU Rooms)	No. of ICU Common Area Samples	% Contaminated ICU Common Areas	Virus Culture Done
Current paper	275	13.1	Yes	275	20	200	14	75	10.7	Partially, negative
Cheng et al *Infect Control Hosp Epidemiol* 2020^19^	377	5.0	Yes	Not stated	1	Not stated	Not stated	0	…	No
Chia et al *Nat Commun* 2020^13^	245	26.5	Yes	35	3	35	0	0	…	No
Colaneri et al *Clin Microbiol Infect* 2020^20^	26	7.7	Yes^a^	Not stated	1	Not stated	Not stated	2	0	Yes, all negative
Guo et al *EID* 2020^15^	252	15.1	Yes	131	Not stated	75	44	5	0	No
Lei et al *Influenza Other Respir Viruses* 2020^39^	400^b^	2.5	Yes	218^b^	4	Not stated	05	0	…	No
Razzini et al *Sci Total Environ* 2020^40^	37	24.3	Yes	12	2	12	41.7	21	19	No
Ryu et al *Am J Infect Control* 2020^17^	79	16.5	Yes	23	2	23	26.1	0	…	No
Su et al *J Microbiol Immunol Infect* 2020^29^	117	1.7	Yes	39^c^	1	39	5.1	0	…	No
Wu et al *Am J Infect Control* 2020^14^	200	19.0	Yes	24	Not stated	24^d^	37.5	Not stated^d^	NA	No
Ye et al *J Infect* 2020^16^	626	13.6	Yes	69	Not stated	69^d^	31.9	Not stated^d^	NA	No
Zhou et al *Clin Infect Dis* 2020^38^	218	52.3	Yes	35	0	…	NA	35	8.6	Yes, all negative
Colaneri et al *J Hosp Infect* 2020^41^	16	0	No	NA	NA	NA	NA	NA	NA	No
Hu et al *Sci Total Environ* 2020^42^	23^e^	47.8	No	NA	NA	NA	NA	NA	NA	No
Ong et al *JAMA* 2020^12^	140	12.1	No	NA	NA	NA	NA	NA	NA	No
Jerry et al *J Hosp Infect* 2020^43^	56^e^	21.4	No	NA	NA	NA	NA	NA	NA	No
Shin et al *Infect Control Hosp Epidemiol* 2020^44^	24	0	No	NA	NA	NA	NA	NA	NA	No
Wang et al *Int J Infect Dis* 2020^14^	36	0	No	NA	NA	NA	NA	NA	NA	No
Wang et al *J Hosp Infect* 2020^45^	84	7.1	No	NA	NA	NA	NA	NA	NA	No
Wee et al *Am J Infect Control* 2020^46^	445	2.2	No	NA	NA	NA	NA	NA	NA	No
Wei et al *mSphere* 2020^47^	112	39.3	No	NA	NA	NA	NA	NA	NA	No

Note. ICU, intensive care unit; NA, not applicable.

a Sampling was done in a “sub-intensive care unit” and emergency unit, and individual numbers were not reported.

b Total number of samples for both air and surface samples; exact number of surface samples not specified.

c One sample, the inside of a closed suctioning tube, was excluded as we did not consider this an environmental sample.

d Not stated in paper whether ICU samples were divided into rooms are common areas. Percentage reported is percentage positivity of all ICU samples.

e Only samples taken before environmental decontamination were included.

## Discussion

In this study, we report the presence and extent of environmental contamination by SARS-CoV-2 in a dedicated COVID-19 ICU. The overall contamination rate was low, and there was no difference in environmental contamination between those on mechanical ventilation or HFNO compared to those on room air. We also found limited contamination of the ICU common areas outside patient rooms.

Compared to other environmental sampling studies (Table 4), the degree of environmental contamination in the ICU was lower in our cohort, with 14% of patient room samples testing positive compared to a median of 29% (range, 0–44%) in the other 6 studies from which data were available. However, variation in sampling technique, patient profile, environmental ventilation settings, cleaning methods, and study design limits direct comparisons with other studies. Compared to an earlier study at our center that utilized the same standardized sampling protocol,^13^ the extent of environmental contamination in the ICU was lower than in the general wards (with overall 26.5% of collected samples testing positive).

The lower extent of environmental contamination seen in the ICUs could be due to several reasons. First, viral shedding has been reported to peak in the first week of illness and to decrease thereafter,^21,22^ which coincides with the time in which most patients develop respiratory complications necessitating ICU admission.^24,25^ The median day of illness (day 14) during sampling in our cohort is consistent with this. Second, patients in the ICU are confined to their bed and unable to walk around the room, thus reducing the chance of direct or indirect droplet spread. The patient with the greatest contamination in our study (50% of surfaces contaminated) was not requiring ventilatory support and was ambulant. Third, closed ventilatory circuits in mechanically ventilated patients likely limit the extent of aerosol or droplet dispersion from respiratory secretions. Su et al^29^ tested environmental samples around 3 patients including 1 ICU patient, and although all environmental samples were negative, swabs taken from inside the ventilation and closed suction tubings were positive, supporting the hypothesis that the closed-loop ventilatory systems prevent environmental contamination.

Increased surface contamination was not associated with mechanical ventilation or HFNO, which suggests that such ventilatory modalities do not enhance SARS-CoV-2 viral dispersion.^30^ Although we did not directly measure aerosol or droplet generation, surface contamination may be used as a surrogate in assessing the extent of such generation because aerosols and droplets are deposited on environmental surfaces via gravity. In vitro studies using manikins and smoke dispersion have found that HFNO did not increase dispersion distance compared to simple oxygen or Venturi masks.^31,32^ HFNO was also not associated with increased environmental contamination for bacterial pneumonia in a randomized–controlled trial comparing its use with conventional oxygen masks.^33^ Our results add to the data supporting the use of HFNO in hypoxemic respiratory failure in COVID-19 from an infection control perspective.

We did not find AGPs to be associated with increased environmental contamination, though only 3 patients underwent AGPs in the 24-hour window prior to sampling, and this small sample size limits conclusive interpretation. AGPs should still be considered high-risk procedures in terms of infection transmission. Novel engineering solutions, such as protective aerosol barriers or hoods, have been proposed to limit the aerosol and droplet dispersal associated with AGPs.^34,35^ However, there has been concern regarding breach of PPE and delayed intubation times associated with some of these contraptions, and their routine use cannot be recommended until more data emerge.^36^

The contamination of surfaces in the common area and staff lounge, while unexpected, is likely to be of low impact in terms of infection transmission risk. The Ct values were high and close to the limit of detection, meaning that the amounts of nucleic acid detected were minute. A lower Ct value has been shown to correlate with successful isolation in viral culture,^21,37^ with a cutoff of 24 in a study in which clinical samples were assessed.^37^ Zhou et al^38^ have also demonstrated in in vitro studies that inoculated environmental samples with a Ct value >30 would not be positive on culture. Consistent with this, we were unable to isolate virus from these specimens. Similar to our findings, contamination of common areas, including water dispenser buttons and desktop computers, was also reported by Wu et al.^14^ Small amounts of nucleic acid could have been deposited on surfaces outside patient rooms through cross contamination after contact with the floor, shoes, or other fomites exiting patient rooms. Although the risk of infection from contact with such contaminated surfaces is infinitesimally small, attention should nonetheless be given to rigorous infection control precautions, decontamination protocols, and strict hand hygiene.

This study has several limitations. First, we could not perform viral culture on all samples that tested positive by PCR due to resource limitations, and we tested only a subset of positive samples from the common area and staff pantry, as we considered the downstream implications on infection control policy to be greater if this contamination was viable virus. PCR positivity alone for the samples taken from the patient rooms does not equate to infective virus, and the PCR assays may have detected nonviable viral nucleic acid. However, because we compared environmental contamination across various patient groups, RNA contamination may act as an acceptable outcome measure. Second, the sample size was small with only 20 patient rooms sampled; thus, this study may have been underpowered to detect smaller differences with regard to patient or disease factors affecting the degree of environmental contamination. Third, there were no patients receiving NIV; hence, we could not assess the potential impact of NIV.

In conclusion, environmental contamination was seen in the ICU, both in patient rooms and common areas. Contamination did not differ depending on the mode of ventilatory support, supporting the safe use of HFNO from an infection control perspective. The frequency and extent of contamination in the ICU was lower compared to general ward settings. Although the infectious risk of horizontal transmission from contaminated surfaces is low, attention should be given to the maintenance of strict hygiene, decontamination, and infection control precautions.

## Supplementary material

For supplementary material accompanying this paper visit https://doi.org/10.1017/ice.2020.1278. click here to view supplementary material
