# Supplementary material for: Environmental contamination in a coronavirus disease 2019 (COVID-19) intensive care unit—What is the risk?
Source: Infect Control Hosp Epidemiol. 2020 Oct 21:1–9. doi: 10.1017/ice.2020.1278 (PMC7653228; doi:10.1017/ice.2020.1278)
Supplement: Supplementary file 1 [file S0899823X20012787sup001.docx]

**Supplementary Online Material – Ong et al., “Environmental contamination in a COVID-19 intensive care unit (ICU) – what is the risk?”**

**Contents:**

1. Supplementary Figure 1: Layout of ICU room depicted surfaces sampled.
2. Supplementary Figure 2: Layout of common area in the ICU depicted surfaces sampled.


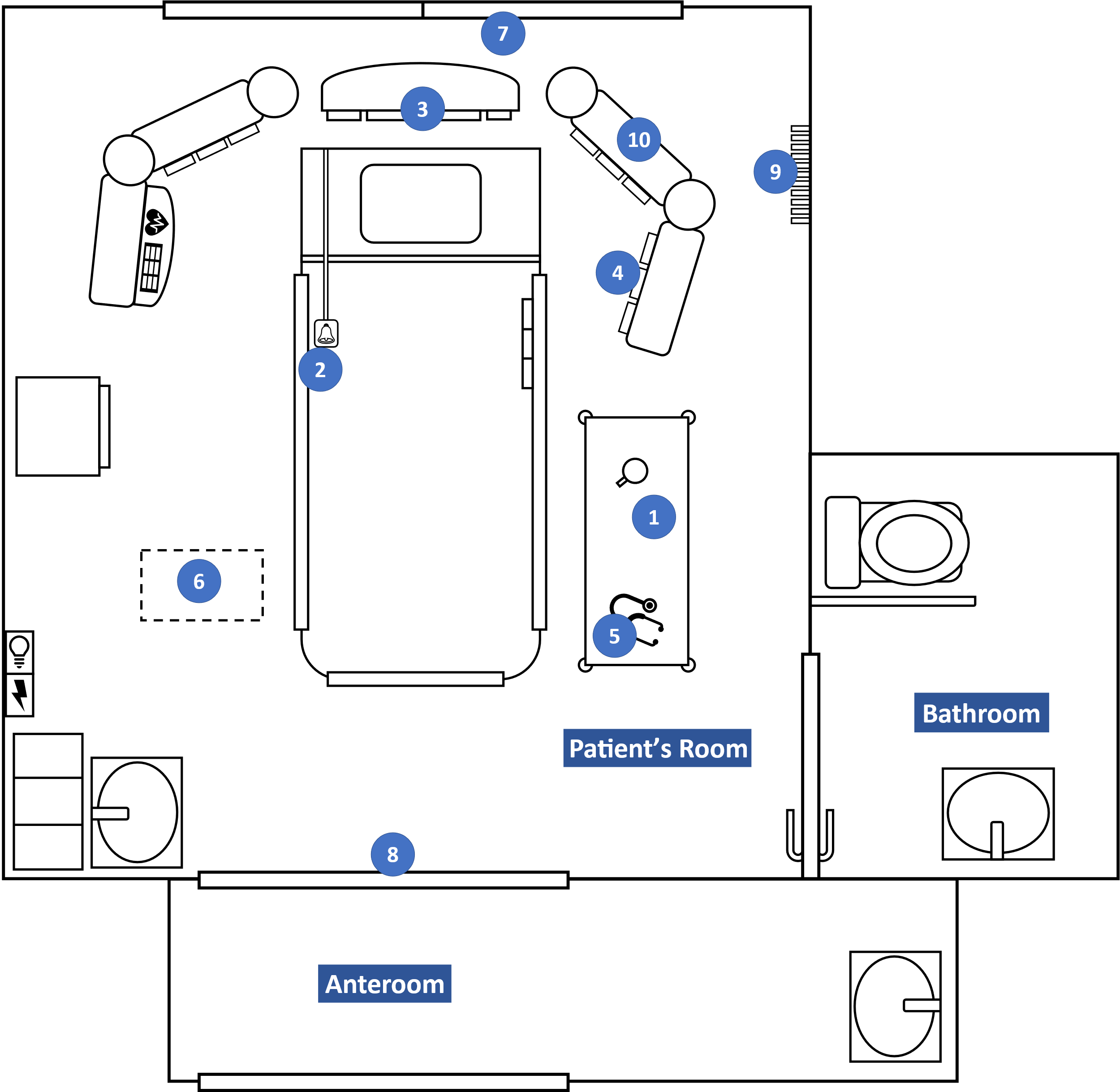
**Supplementary Figure 1:** **Layout of ICU room depicted surfaces sampled.**

Blue circles indicate surfaces sampled: (1) cardiac table, (2) bed rail including call bell, (3) ventilator or high-flow nasal oxygen machine*, (4) medication infusion pumps, (5) stethoscope, (6) floor, (7) glass window, (8) glass door, (9) air outlet vent, and (10) surgical pendant.

*For patients not on invasive ventilation and not receiving high-flow nasal oxygen, the corresponding area on the back of the bed was swabbed instead.

**
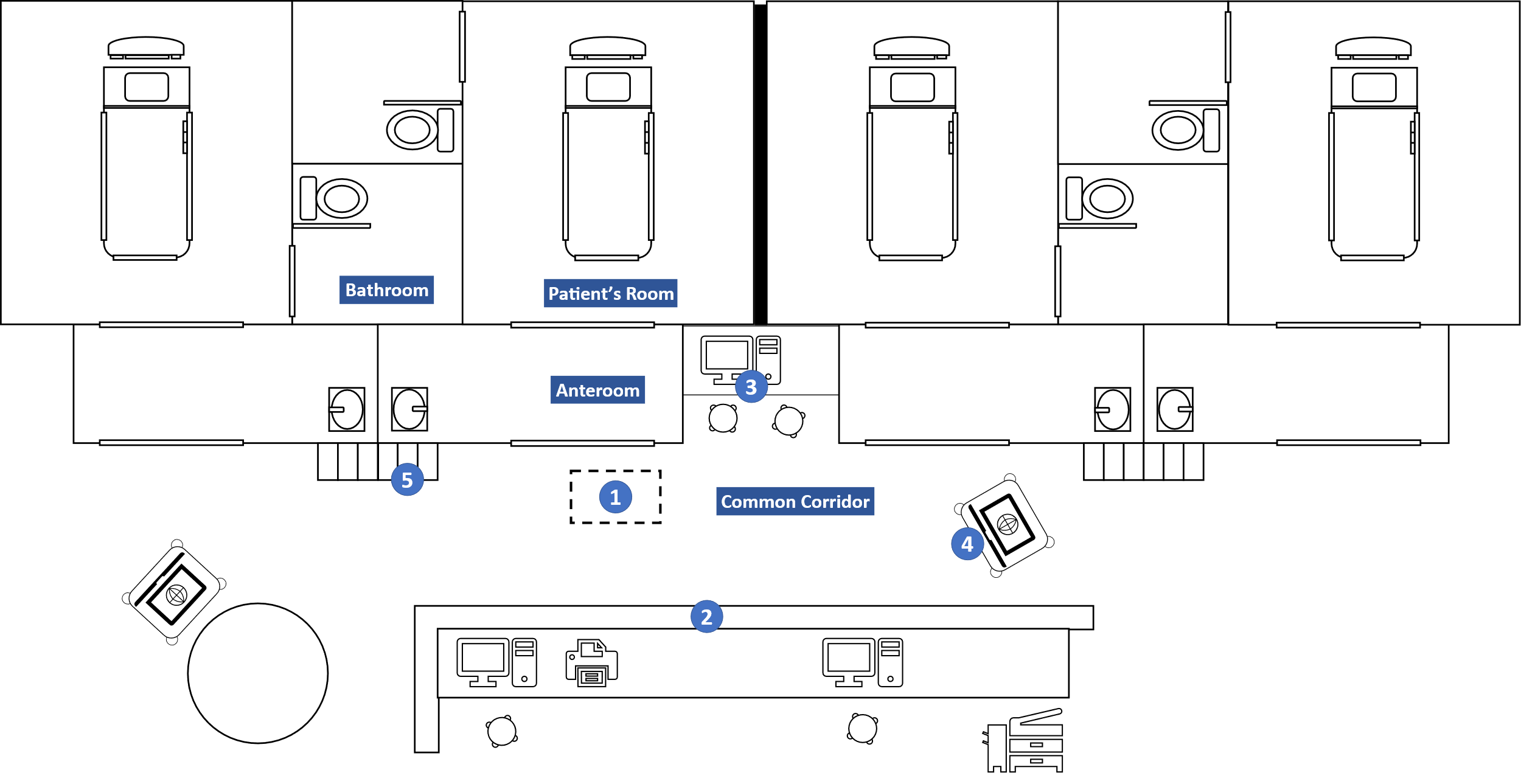
Supplementary Figure 2:** **Layout of common area in the ICU depicted surfaces sampled.**

Blue circles indicated surfaces sampled: (1) floor, (2) nursing counter, (3) desktop computer including keyboard and mouse, (4) mobile computer on wheels, and (5) personal protective equipment storage area
